# Impact of stromal microenvironment on metabolic phenotypes in breast cancer: evidence for stroma-influenced Warburg effect

**DOI:** 10.1186/1753-6561-6-S3-P8

**Published:** 2012-06-01

**Authors:** Heather Ann Brauer, Liza Makowski, Katherine A Hoadley, Lindsay J Lang, Alex J Freemerman, Charles M Perou, Melissa A Troester

**Affiliations:** 1 Department of Epidemiology, Gil lings School of Global Public Health, University of North Carolina at Chapel Hill, Chapel Hill, NC 27599, USA; 2 Department of Nutrition, Gillings School of Global Public Health, University of North Carolina at Chapel Hill, Chapel Hill, NC 27599, USA; 3 Lineberger Comprehensive Cancer Center, University of North Carolina at Chapel Hill, Chapel Hill, NC 27599, USA; 4 Department of Pathology and Laboratory Medicine, School of Medicine, University of North Carolina at Chapel Hill, Chapel Hill, NC 27599, USA

## Background

Cancer cells have altered metabolism, with an increased rate of glucose uptake, increased glycolysis, and due to the Warburg phenomenon, increased production of biomass (amino acids and nucleic acids) as metabolic byproducts. Association of these metabolic changes with breast cancer subtype and tumor gene expression has not been evaluated, nor is it well understood what biological processes and characteristics are most important in driving altered metabolic state in breast cancer.

## Experimental design

Thirty-four breast tumors and six samples of cancer-adjacent normal tissue were analyzed by liquid and gas chromatography (LC/GC) mass spectrometry (MS) to determine the expression of 379 metabolites. The same tissues were also used to perform gene expression microarrays and the resulting gene expression profiles were used to identify tumor subtype. These data combined with clinical tumor characteristics were evaluated in association with metabolic phenotype. To complement these analyses of human tissues, in vitro assays using human breast cancer cells alone and in coculture with cancer-associated fibroblasts (CAFs) were performed to study glucose uptake and utilization.

## Results

Unsupervised cluster analysis on all metabolites resulted in two main clusters, with differences in the prevalence of aggressive tumor subtypes in each cluster. Characteristic of the Warburg phenomenon, four groups of metabolites showed differential levels between the two tumor clusters: amino acids, sugars, nucleic acids, and citric acid (TCA) cycle metabolites. Normal breast tissue samples were similar to less aggressive tumors with low biomass production and decreased levels of glycolysis/TCA byproducts. Parallel to these results in human tissues, glucose uptake was increased in basal-like breast cancer cells when they were cocultured with fibroblasts, but less aggressive luminal-fibroblast cultures did not show increased glucose uptake. Rather, in luminal cocultures, the fibroblasts appeared to suppress glucose uptake. Glucose utilization (oxidation and glycogen synthesis) was also higher in basal-like mono- and cocultures compared to luminal cultures. Consistent with these findings that the stroma influences metabolic phenotype, tumors that had active wound response gene expression had the more aggressive, Warburg-like metabolic profile.

## Conclusions

We observed distinct metabolic profiles associated with more aggressive breast cancer tumors. Based both on the observed patterns of glucose uptake and utilization in cocultures, and on the association between wound response activation and metabolic profile, these metabolic signatures appear to be influenced by multiple factors, including the stroma. These data suggest that integration of genomic and metabolic data provide insights for better understanding a Warburg effect in human breast cancer that may be regulated by the stroma.

